# Pre-Ischemic Treadmill Training Induces Tolerance to Brain Ischemia: Involvement of Glutamate and ERK1/2

**DOI:** 10.3390/molecules15085246

**Published:** 2010-08-02

**Authors:** Feng Zhang, Yi Wu, Jie Jia, Yong-Shan Hu

**Affiliations:** Department of Rehabilitation Medicine, Hua Shan Hospital, Fudan University, WuLuMuQi Middle Road 12, Shanghai 200040, China; E-Mail: zjk20019@126.com (F.Z.)

**Keywords:** ERK1/2, exercise-induced neuroprotection, glutamate, ischemia/reperfusion injury, microdialysis

## Abstract

Physical exercise has been shown to be beneficial in stroke patients and animal stroke models. However, the exact mechanisms underlying this effect are not yet very clear. The present study investigated whether pre-ischemic treadmill training could induce brain ischemic tolerance (BIT) by inhibiting the excessive glutamate release and event-related kinase 1/2 (ERK1/2) activation observed in rats exposed to middle cerebral artery occlusion (MCAO). Sprague–Dawley rats were divided into three groups (n = 12/group): sham surgery without prior exercise, MCAO without prior exercise and MCAO following three weeks of exercise. Pre-MCAO exercise significantly reduced brain infarct size (103.1 ± 6.7 mm^3^) relative to MCAO without prior exercise (175.9 ± 13.5 mm^3^). Similarly, pre-MCAO exercise significantly reduced neurological defects (1.83 ± 0.75) relative to MCAO without exercise (3.00 ± 0.63). As expected, MCAO increased levels of phospho-ERK1/2 (69 ± 5%) relative to sham surgery (40 ± 5%), and phospho-ERK1/2 levels were normalized in rats exposed to pre-ischemic treadmill training (52 ± 6%) relative to MCAO without exercise (69% ± 5%). Parallel effects were observed on striatal glutamate overflow. This study suggests that pre-ischemic treadmill training might induce neuroprotection by inhibiting the phospho-ERK1/2 over-activation and reducing excessive glutamate release

## 1. Introduction

Brain ischemic tolerance (BIT) is a phenomenon in which resistance to cerebral ischemia is induced by harmful stimuli that are below the threshold causing cell damage [1]. Multiple stimuli have been studied, including hypoxia [2], ischemia [1], anoxia [3], oxidative stress [4], and inhibitors of oxidative phosphorylation [5]. However, patients may be adverse to such prophylactic treatments, and finding more attractive treatments has recently become an important research focus. Several reports indicate that protective effects can be induced by normobaric hyperoxia [6], electro-acupuncture [7], and pre-ischemic treadmill training [8], among others.

During the past 20 years, the beneficial effects of physical exercise on stroke injury have been demonstrated, including reduction of neurologic deficits, amelioration of blood–brain barrier (BBB) dysfunction, maintenance of neurovascular integrity and promotion of enhanced survival [9,10,11,12,13,14]. In addition, a previous paper demonstrated that two or four weeks of treadmill pre-training was neuroprotective for ischemia, decreasing both cerebral infarction and edema [15] 

After a transient ischemic attack, excessive glutamate release could evoke postsynaptic depolarization and cause cation inﬂux into the cells, possibly inducing neuronal excitotoxicity [16,17]. Moreover, neuroprotection induced by oxygen/glucose deprivation enhances reuptake of excessive extracellular glutamate through excitatory amino acid transporters (EAATs) and glutamate transporters [18,19]. Furthermore, we recently reported that pre-ischemic treadmill training regulated the concentration of glutamate and gamma-aminobutyric acid (GABA) after ischemic stroke [20]. A previous study showed an improvement in survival and a marked reduction in brain damage in gerbils subjected to 2-week preischemic locomotor activity [21]. A recent study also showed a 3-week preischemic treadmill training could promote BBB function and enhance basal lamina integrity for Sprague–Dawley rats subjected to ischemic stroke [22]. However, whether 3-week pre-ischemic treadmill training could influence the excessive release of glutamate caused by cerebral ischemia is still unknown. 

The event-related kinase 1/2 (ERK1/2) pathway has been shown to play a key role in the neuroprotective effect of pre-ischemic exercise [8]. ERK1/2 belongs to an important subfamily of mitogen-activated protein kinases that control a broad range of cellular activities and physiological processes, including reactions to cerebral ischemia [23]. Inhibition of the ERK1/2 pathway has been shown to reduce brain damage and focal infarct volume in mice after ischemia [24,25]. Additionally, a recent study demonstrated that pre-ischemic exercise can alleviate ischemic damage by regulating the expression of HSP-70 and phospho-ERK1/2 [8]. Thus, the present study investigated the effect of three weeks of pre-ischemic exercise on neurological signs, infarct size, phospho-ERK1/2, and glutamate.

## 2. Results and Discussion

### 2.1. Physiological parameters

Body temperature, heart rate, blood gases and pH, blood pressure were similar in three groups before, during and after the process of *in vivo* microdialysis and ischemia/reperfusion. All animals completed the study.

### 2.2. Behavioral scores

**Figure 1:**
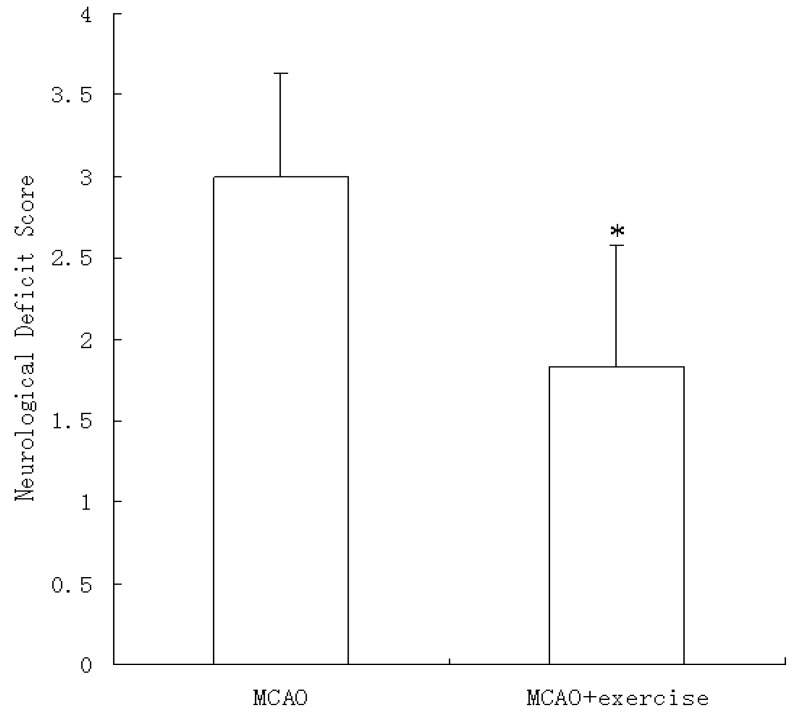
Neurological deficits at 48 h after reperfusion with or without prior exercise. MCAO caused neurological deficits that were not observed in sham surgery animals. Three weeks of exercise prior to MCAO significantly decreased neurological deficits. Data represent median ± interquartile range. *P < 0.01 relative to MCAO.

No neurological symptoms were observed prior to surgery or 48 h after sham surgery (data not shown). However, at 48 h after reperfusion, neurological signs were apparent (3.00 ± 0.63), and pre-MCAO exercise significantly decreased this effect (1.83 ± 0.75, P < 0.05), as shown in Figure 1. 

### 2.3. Infarct volume

**Figure 2:**
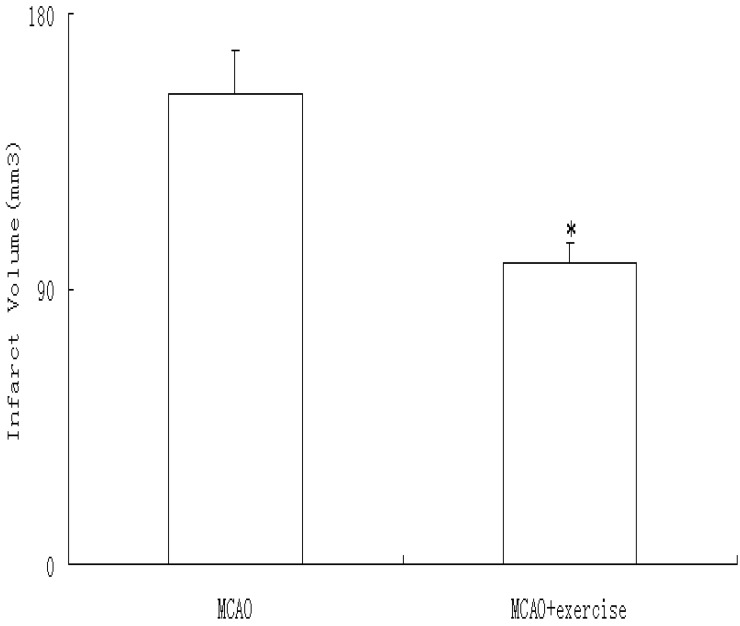
Cerebral infarction volume at 48h after reperfusion, was determined via TTC staining. MCAO animals show infarcts, which are decreased by prior exercise. Data represent mean ± SD. * P < 0.05 relative to MCAO.

At 48 h after reperfusion, no infarct was observed in animals exposed to sham surgery, but MCAO caused infarcts (175.9 ±13.5 mm^3^) that were reduced by prior exercise (103.1 ± 6.7 mm^3^, P < 0.05) (Figure 2). 

### 2.4. Glutamate levels

**Figure 3:**
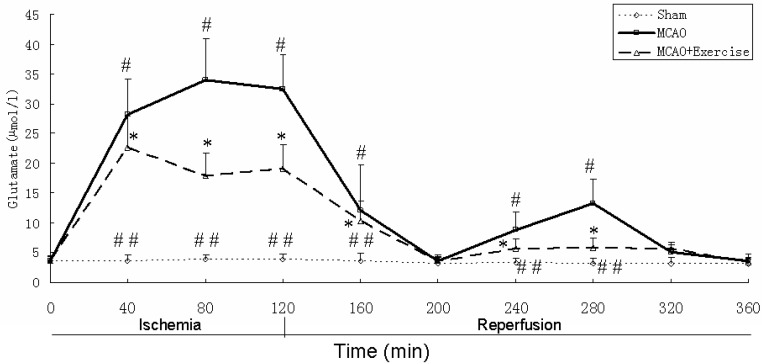
Striatal dialysate glutamate levels. See text for discussion. Data represent mean ± SD. ^#^ P < 0.05 relative to sham surgery at the same time point. ^##^ P < 0.05 relative to MCAO+Exercise at the same time point.* P < 0.05 relative to MCAO at the same time point.

The glutamate concentration was 3.59 µmol/L before ligation (Figure 3). After sham surgery, stable levels of extracellular glutamate were observed throughout the 360-min period. After MCAO, glutamate was elevated and peaked at 80 min (33.69 ± 7.03 µmol/L), decreased to baseline levels at 200 min (80 min after reperfusion began) (3.55 ± 0.77 µmol/L), increased to a second peak at 280 min (160 min after reperfusion began) (13.22 ± 4.13 µmol/L), then returned to baseline again by 320 min. In MCAO animals with prior exercise, three weeks treadmill training suppressed the level of glutamate by 46%, at 80 min, 55% at at 280 min (160 min after reperfusion began) (P < 0.05). This decreased level of gluta-Group differences among the three groups were apparent at most time points, except at 0 (baseline), 200, 320 and 360 min (P < 0.05). 

### 2.5. ERK1/2 and Phospho-ERK1/2

**Figure 4:**
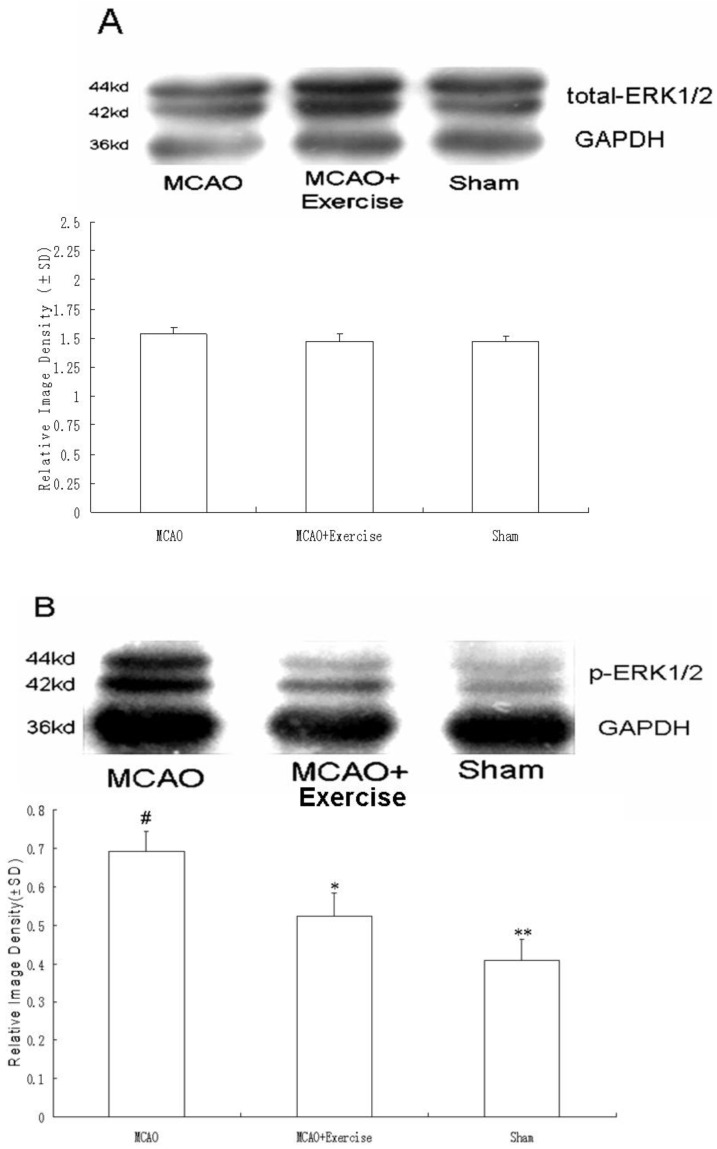
Protein levels of total ERK1/2 and phospho-ERK1/2. A) No significant differences were observed for total-ERK1/2. B) Phospho-ERK1/2 was higher in MCAO than sham surgery and was partially reduced with exercise. Data represent mean ± SD. ^#^ P < 0.05 relative to sham surgery. **P < 0.05 relative to MCAO+Exercise.* P < 0.05 relative to MCAO.

Total ERK1/2 levels were similar among all three groups, as shown in Figure 4. However, phospho-ERK1/2 showed group differences. Specifically, MCAO animals showed increased phospho-ERK1/2 (69 ± 5%, P < 0.05) relative to sham surgery (40 ± 5%), and this effect was reduced by prior exercise (52 ± 6%, P < 0.05).

### 2.6. Discussion

The current data demonstrate that glutamate and ERK1/2 are involved in the brain’s responses to ischemia. Understanding the temporal alterations of these proteins is crucial, as they represent important targets for intervention to improve the functional outcome after ischemia. 

ERK1/2 plays differential roles during the pre- and post-stroke periods. According to previous studies, treatment with a mitogen-activated protein kinase(MAPK)/ERK kinase(MEK1) inhibitor decreases brain damage in mice after ischemia, suggesting that the MEK1-ERK1/2 pathway contributes to cerebral damage during ischemia [24,25]. Thus, excessive phospho-ERK1/2 after stroke is deleterious, and treatments that suppress this effect could decrease ischemia-induced damage.

In a recent study, phosphorylation of ERK1/2 promotion by pre-ischemic treadmill exercise was neuroprotective, and this neuroprotection was blocked by the inhibitor of ERK1/2 [8]. This study also showed that pre-ischemic exercise was neuroprotective by elevating the level of phospho-ERK1/2 [8]. Similar to their paper, we also observed pre-ischemic exercise could alleviate the over-expression of ERK1/2 after ischemic stroke. In addition, because of the similarity in ERK1/2 and glutamate changes before and after stroke, we also measured the glutamate concentration difference.

As the most abundant neurotransmitter in the central nervous system (CNS), glutamate activates mammalian *N*-methyl-D-aspartate (NMDA), α–amino–3–hydroxy–5–methyl-4-isoxazole propionic acid (AMPA), kainite (KA) receptors, and is essential for normal cerebral function [26]. It has been suggested that whether glutamatergic activation is beneficial or harmful depends on the degree of activation, which is influenced by events such as ischemia. For example, a slight up-regulation of glutamate expression was found to have a beneficial effect on memory [27], while an excessive increase in glutamate such as that induced by transient complete cerebral ischemia (e.g., 8-fold increase [28]) has been associated with neuronal excitotoxicity [16,29,30]. Based on these previous studies, several different methods have been investigated to suppress the excessive release of glutamate after transient ischemic attack, including the induction of aminobutyric acid type A (GABA) release [31], AMPA/KA (non-NMDA) receptor antagonists [32], NMDA receptor antagonists [33], and GABA-B receptor agonists [34]. These studies suggest that after cerebral ischemia, downregulation of excessive glutamate release alleviates ischemia-induced damage, while moderate activation of glutamate before cerebral ischemia may be protective for cerebral function.

Moreover, persistent activation of ERK1/2 is associated with glutamate-induced oxidative toxicity in neuronal cells, and inhibition of ERK1/2 activation protects cells from glutamate toxicity [35]. This study implies that there is connection between the activation of ERK1/2 and glutamate.

In the present study, pre-ischemic exercise reduced infarct volume and neurological impairment after MCAO, demonstrating that pre-ischemic treadmill training could induce BIT. Furthermore, pre-ischemic exercise alleviated the excessive increase in glutamate levels after MCAO. Excessive glutamate could cause severe brain damage through calcium overload, production of oxidative species, depletion of ATP, and other processes. Similarly, phospho-ERK1/2 levels showed the same pattern as glutamate, but there was no effect on total ERK1/2. These results suggest that cerebral ischemia and exercise do not change ERK1/2 at the gene expression level, but pre-ischemic treadmill training can down-regulate the ischemia-induced increase of phospho-ERK1/2 and effectively protect the nervous system.

## 3. Experimental

### 3.1. Subjects

Thirty-six male Sprague-Dawley rats, weighing 250–300 g at the beginning of the experiment, were obtained from the Shanghai Laboratory Animal Center, Chinese Academy of Sciences. The rats were maintained under a 12-h light/dark cycle with food and water available *ad libitum*. All procedures were approved by the Animal Care and Use Committee of Fudan University. 

### 3.2. Treadmill training

Rats were randomly assigned to one of three groups (n = 12/group): sham surgery without prior exercise, MCAO without prior exercise and MCAO with prior exercise. Before training, rats in the exercise group underwent two days of adaptive running exercise at a speed of 5–8 m/min for 30 min/day on an electric treadmill machine (DSPT-202 Type 5-Lane Treadmill; Litai Biotechnology Co., Ltd, China). After the adaptive exercise period, the rats received three weeks of treadmill training at a speed of 20 m/min, 30 min/day for six days per week. The rats in the sham surgery without exercise and MCAO without exercise groups did not receive treadmill training but were allowed to run freely in their cages for the three weeks. 

### 3.3. MCAO model

Once treadmill training was complete, rats underwent stereotaxic surgery. They were anesthetized with 12% (v/v) chloral hydrate (0.345 mg/kg, i.p.) and were maintained under anesthesia for as long as 6 h by gradually supplementing chloral hydrate as necessary. Each rat was fixed in a stereotaxic apparatus, and a hole was incised in the skull (relative to bregma: AP -0.4 mm, ML +3.4 mm). A catheter with a stainless steel cannula was inserted into the striatum (AP -0.4 mm, ML +3.4 mm, DV -3.0 mm) and ﬁxed to the skull with dental cement, anchored by two screws. The left MCA was occluded using the intraluminal suture method [36] with minor modiﬁcations. Briefly, the right common carotid artery, internal carotid artery, and external carotid artery were exposed by surgical operation. A 4-0 monofilament nylon suture (Beijing Sunbio Biotech Co., Ltd, Beijing, China.) with a round poly-L-lysine coated tip was precisely inserted into the external carotid artery. Next, the suture direction was changed toward the internal carotid artery, and it was gently advanced to occlude the MCA. The body temperature of rat was maintained at 37 ºC with a heating pad. After 120 minutes, the suture was removed from the internal carotid artery. In the sham group, the external and internal carotid arteries underwent the same procedure without the last step of MCAO. Physiological parameters were obtained by a Blood Gas and Electrolyte System (Radiometer ABL505, Copenhagen, Denmark). Rats were evaluated 48 hours after reperfusion according to a 5-point scale [36] as follows: 0, no neurological symptoms; 1, unable to completely extend the front jaw on the contralateral side; 2, rotating while crawling and falling to the contralateral side; 3, unable to walk without assistance; and 4, unconsciousness. 

### 3.4. *In vivo* microdialysis

Each rat was anesthetized with 12% (v/v) chloral hydrate and fixed in the prone position in a stereotaxic apparatus. A balanced microdialysis probe (15 kD cut-off PES membrane, 4 mm long; MAB6.14.4 probe; Bioanalytical System, Inc., West Lafayette, IN, USA) was inserted into the striatum via the previously implanted cannula. The inlet tube of the microdialysis probe was linked to a microinfusion pump (MD-0100; Bioanalytical System), and the outlet tube was linked to a 0.5 mL centrifuge tube to collect dialysate for later quantification of glutamate. The procedure was similar to the procedure reported in the present manuscript [20]. Samples were obtained before ischemia, at 40, 80 and 120 min after ischemia began, and at 40, 80, 120, 160, 200 and 240 min after ischemia ended and reperfusion began.

### 3.5. Amino acid measurement by HPLC

An Agilent 1100 LC system (Palo Alto, CA, USA) and HPLC workstation (Hewlett Packard, Palo Alto, CA, USA) were used for measuring the concentration of amino acids. Stock solutions of each amino acid standard (10 mM) were prepared in 100 mM potassium carbonate in 50% (v/v) methanol. Amino acid standards or samples (20 μL) were derivatized by *o*-phthalaldehyde (OPA). An excellent linear curve was obtained over the range 0.5–100 μM for each the detecting amino acid (R^2^ > 0.999). The concrete procedure was shown in our previous paper [20].

### 3.6. Determination of brain infarct volume

At 48 h after reperfusion, animals were decapitated under 12% (v/v) chloral hydrate anesthesia. Brains were stored at −20 ºC for 10 min, then six coronal sections (2 mm thick) were dissected beginning mid-way between the anterior pole and the optic chiasm and proceeding caudally. All sections were immediately placed into a 2% TTC (2,3,5-triphenyltetrazolium chloride) solution at 37 ºC for 30 min, then ﬁxed in 4% paraformaldehyde buffer. After 24 hours, slices were photographed with a digital camera (DC240; Kodak, USA) and imaging software (Adobe Photoshop 7.0) was used to calculate the area of infarction. The total infarction volume was measured as the sum of the infarcts of each of the six sections. To minimize the error caused by brain edema, the corrected infarct volume was calculated as follows: infarct area X {1 - [(ipsilateral hemisphere area - contralateral hemisphere area)/contralateral hemisphere area]}[37].

### 3.7. Western blot

The cortex surrounding the ischemic zone was dissected. Protein was extracted by protein extraction reagen (Pierce Biotechnology, IL, USA), and protein concentration was determined using the bicinchoninic acid assay. Equal volumes of protein extract (40 µg) and sample buffer were mixed and boiled 5 min at hot water(95 ºC) before loading onto 10% polyacrylamide gels. Proteins were transferred onto Hybond nylon membrane (Amersham, Piscataway, NJ, USA) in 350 V for 2.5 h with the cold pack, incubated in 5% BSA blocking solution 1 hr at room temperature , and immersed overnight at 40 ºC in anti-ERK1/2 (1:1000 dilution; Cell Signaling, Danvers, MA, USA) or anti-phospo-ERK1/2 antibody (1:1000 dilution; Cell Signaling). Horseradish peroxidase-labeled anti-rabbit secondary antibody (1:100 dilution; Hua-Mei Biotech, Beijing, China) was used to detect the immunoreactivity for 1 h at room temperature with blocking buffer. Lastly, the membrane was developed by exposure to 300 µL of enhanced ECL kit (Amersham Pharmacia Biotech, Freiburg, Germany) for 5 min and exposed to Kodak film for 5 to 30 seconds. GAPDH was used as a loading control.

### 3.8. Statistical analysis

Statistical analysis was performed using SPSS for Windows, version 11.0 (SPSS Inc, Chicago, IL, USA). Neurological signs and infarct volume in ischemic rats with or without pre-ischemic exercise were compared by an independent *t*-test. The differences of glutamate concentration in single time point and relative image density among three groups were analyzed using one-way analysis of variance (ANOVA) in single time point. Tukey's post hoc test was used to determine pairwise comparisons. Data are presented as median (interquartile range) for ordinal data and means ± SD for continuous variables. All statistical assessments were two-sided and evaluated at the 0.05 level of signiﬁcance. 

## 4. Conclusions

Overall, our data demonstrate that pre-ischemic treadmill training can exert a protective effect against nervous system damage by inhibiting the increase in glutamate and phospho-ERK1/2 after cerebral ischemia, producing BIT. Based on the assumption that the relationship between glutamate and ERK1/2 may be similar to that between the overload of calcium and oxidative damage after cerebral ischemia/reperfusion, we hypothesize that glutamate and ERK1/2 could interact to exacerbate cerebral ischemic injury. Further studies should investigate the possible involvement of specific glutamate receptors and transporters in the neuroprotection induced by pre-ischemic exercise, but such exercise appears to provide neuroprotection for stroke.
